# A New Cre Driver Mouse Line, Tcf21/Pod1-Cre, Targets Metanephric Mesenchyme

**DOI:** 10.1371/journal.pone.0040547

**Published:** 2012-07-06

**Authors:** Yoshiro Maezawa, Matthew Binnie, Chengjin Li, Paul Thorner, Chi-Chung Hui, Benjamin Alman, Makoto Mark Taketo, Susan E. Quaggin

**Affiliations:** 1 The Samuel Lunenfeld Research Institute, Mount Sinai Hospital, Toronto, Ontario, Canada; 2 Division of Respirology, St. Michael's Hospital, University of Toronto, Toronto, Ontario, Canada; 3 Department of Pediatric Laboratory Medicine, The Hospital for Sick Children, Toronto, Ontario, Canada; 4 Program in Developmental and Stem Cell Biology, The Hospital for Sick Children, Toronto, Ontario, Canada; 5 Division of Orthopaedic Surgery, University of Toronto, Toronto, Ontario, Canada; 6 Department of Pharmacology, Graduate School of Medicine, Kyoto University, Kyoto, Japan; 7 Division of Nephrology, St. Michael's Hospital, University of Toronto, Toronto, Ontario, Canada; 8 Department of Medicine, University Health Network, University of Toronto, Toronto, Ontario, Canada; Institut National de la Santé et de la Recherche Médicale, France

## Abstract

Conditional gene targeting in mice has provided great insight into the role of gene function in kidney development and disease. Although a number of Cre-driver mouse strains already exist for the kidney, development of additional strains with unique expression patterns is needed. Here we report the generation and validation of a *Tcf21/Pod1-Cre* driver strain that expresses Cre recombinase throughout the condensing and stromal mesenchyme of developing kidneys and in their derivatives including epithelial components of the nephron and interstitial cells. To test the efficiency of this line, we crossed it to mice transgenic for either loss or gain of function *β-catenin* conditional alleles. Mice with deletion of *β-catenin* from Tcf21-expressing cells are born with hypoplastic kidneys, hydroureters and hydronephrosis. By contrast, *Tcf21-Cre* driven gain of function for *β-catenin* in mice results in fused midline kidneys and hypoplastic kidneys. Finally, we report the first renal mesenchymal deletion of *Patched1 (Ptch1)*, the receptor for sonic hedgehog (Shh), which results in renal cysts demonstrating a functional role of Shh signaling pathway in renal cystogensis. In summary, we report the generation and validation of a new Cre driver strain that provides robust excision in metanephric mesenchyme.

## Introduction

The introduction of gene targeting in mouse embryonic stem cells in 1985 provided an efficient means to determine the function of gene(s) in mammalian tissues in vivo. More recent advances have permitted cell specific and temporal regulation of gene deletion and overexpression, providing investigators more refined tools to study the role of genes and molecular pathways in specific organs and tissues. The Cre-loxP system is most broadly used, largely due to the availability of a wide variety of floxed mouse lines in public consortia, pharmaceutical companies and academic labs [1].

In order to take full advantage of available floxed lines, renal researchers must be able to choose from a wide variety of complementary, robust and unique Cre-driver strains. Metanephric mammalian kidneys derive from two cell lineages: metanephric mesenchyme and ureteric bud that both arise from the intermediate mesoderm [2]. The metanephric mesenchyme is comprised of subpopulations including the self-renewing cap mesenchyme and stromal mesenchyme. The cap mesenchyme gives rise to all of the epithelial cell derivatives of the nephron from the glomerulus to the distal nephron, whereas the stromal mesenchyme is believed to give rise to interstitial cells such as myofibroblasts and pericytes, although the precise molecular steps have not been determined [3] [4]. A number of Cre driver strains exist for early kidney studies; e.g. *HoxB7* [5] for the ureteric bud lineage, *Six2* and *Pax3* for the cap mesenhcyme [4], [6], [7], *Foxd1* for the stromal population [8] and *Wnt4* for the renal vesicles [9]. In addition, a number of Cre driver strains exist for fully differentiated cells in the nephron (reviewed in [2]).

Tcf21/Pod1 is a basic helix loop helix transcription factor with a unique expression pattern in both cap and stromal mesenchyme, providing an opportunity to develop a Cre-driver strain capable of broad deletion in metanephric mesenchymal populations [10], [11], [12]. Here we report the generation of a Cre-driver line using the endogenous *Tcf21* promoter and describe robust renal phenotypes when this Cre driver strain is crossed to mice carrying a floxed *β-catenin* allele causing loss of function, an allele resulting in *β-catenin* gain of function and a floxed *Ptch1* receptor allele. The *Tcf21-Cre* line provides a robust and valuable alternative genetic tool for the developmental renal biologist.

## Materials and Methods

### Ethics Statement

All mouse experiments were approved by the animal ethics committee at the Toronto Center for Phenogenomics, and were performed in accordance with ‘Canadian Council of Animal Care’ regulations.

### Construction of a *Tcf21-Cre* targeting vector

To make the *Tcf21-Cre* targeting vector, a 3.1 kb fragment including intron 1, exon 2 and the 3′ UTR of the *Tcf21* gene (accession number AF296764) was amplified from murine genomic DNA by PCR and subcloned into the KpnI site of the pKO (knockout) vector (a kind gift from Dr. J. Rossant, The Hospital for Sick Children, Toronto, Canada). For the 5′ homology arm, a 3.5 kb fragment was amplified from the 5′ flanking region of exon 1, subcloned into pBluescript KS+, and excised using SmaI and SalI. The Cre recombinase cassette and β-actin polyA fragment were excised from the NLS-Cre plasmid (kind gift from Dr. Brian Sauer, Oklahoma Medical Research Foundation, Oklahoma City, OK) and Nephrin promoter NLS-Cre plasmid [13] using EcoRI/SalI sites and EcoRI/XbaI sites, respectively. The *Tcf21-PKO* vector was digested with XbaI and PmlI, and the four fragments were subjected to ligation to construct the final *Tcf21-Cre* targeting vector.

### ES cell culture and generation of chimeras

The *Tcf21-Cre* vector was linearized by Pme1 and electroporated into murine R1 ES cells derived from male blastocyst, hybrid of two 129 substrains (129X1/SvJ and 129S1/SV-+^p^+^Tyr-c^Kitl^Sl-J^/+) [14]. After selection with G418 and FIAU (5-Iodo-2′-fluoroarauracil), resistant clones were selected and subjected to Southern analysis using a 3′ *Tcf21* genomic 500 bp probe outside the region of homology that recognized a 5.3 kb and a 9.1 kb HindIII fragment for the mutant and wild-type alleles, respectively. Embryo manipulation and aggregation of the ES cell clones were carried out. One ES cell line generated chimeras that gave germline transmission.

### Breedings of mouse lines

The *Tcf21-Cre* heterozygous founder mice were crossed with a *Z/EG* reporter murine line (kind gift from Dr. A Nagy, the Samuel Lunenfeld Research Institute). The *Z/EG* reporter construct is comprised of a systemic pCAGGS promoter which directs the loxP-flanked β-Geo (lacZ/neomycin-resistance and STOP signal) fusion gene followed by a green fluorescent protein (GFP) cassette [15]. Site-specific Cre recombinase expression leads to removal of the STOP signal resulting in GFP expression. *Tcf21-Cre* mice were then bred to *β-catenin* conditional loss of function (LOF) mice (*Ctnnb1^fl/fl^*) [16], *β-catenin* conditional gain of function (GOF) mice (*Ctnnb1^ex3/+^*) [17], and conditional loss of function *Ptch1* mice (*Ptch1^fl/fl^*) [18] to examine their phenotypes. Six2-Cre mouse line was a kind gift from Dr. Andrew P. McMahon (Harvard University, Cambridge), and crossed with Z/EG mouse line.

### Genotyping

*Tcf21-Cre* mice offsprings were genotyped by 5′mPod-1 primer (5′-CGGGACTGCCAGATCCCACC-3′), AS-mPod-1 primer (5′-CCTGCTTGCCCTCCTGGGTG -3′) and CRE106AS primer (TTCTCCCACCGTCAGTACGTCA) that produce a 353 bp product for the wild type allele and a 719 bp product for the mutant allele, respectively. Genotyping of the LOF and GOF *β-catenin* mice and *Ptch1* mice have been described previously [16], [17], [18].

### Phenotypic analysis

Murine embryos were dissected at specific time points described, and macroscopic phenotypes of each organ were photographed using a Leica dissecting microscope (Leica, MZ6). Whole embryos or organs were fixed in 10% formalin in phosphate-buffered saline (PBS) and subjected to histological analysis.

### Immunostaining

For immunohistochemistry, tissues were dissected and fixed in 10% formalin/PBS and embedded in paraffin. 5 µm-thick sections were then rehydrated, boiled in citrate buffer for antigen retrieval, and endogeneous peroxidases were quenched with 3% hydrogen peroxide. For immunofluorescence, samples were fixed overnight in 4% paraformaldehyde at 4°C, cryoprotected in 30% sucrose overnight, embedded in Tissue-Tek OCT 4583 compound (Sakura Finetek USA Inc., Torrance, CA, USA) and 10 µm sections were cut by cryostat (Leica CM3050S). After blocking (10% goat serum, 3% albumin and 0.3% TritonX100 in PBS), samples were incubated with primary antibodies at 4°C overnight, washed and incubated with secondary antibodies for 1 h at room temperature. Primary antibodies used for this study included anti-GFP (A11122, Invitrogen, Camarillo, CA, USA), anti-alpha-smooth muscle actin (α-Sma, ab5694, Abcam, Cambridge, MA, USA) and anti-Tamm Horsfall protein (BT-590, Biomed Technologies, Inc. Stoughton, MA, USA). Immunohistochemical staining was carried out using Vectastain ABC kit (Vector Laboratories). Diaminobenzidine (Vector Laboratories) was used for the color reaction.

Two lectins were utilized to stain specific renal segments. Biotinylated Dolichos biflorus agglutinin (DBA, B-1035, Vector Laboratories, Burlingame, CA, USA) was used as a specific marker of the collecting duct, and visualized by FITC labeled avidin (Vector Laboratories). FITC labeled Lotus tetragolonobus lectin (LTL, FL-1321, Vector Laboratories) is a specific marker of the proximal tubule and was visualized directly with a fluorescent microscope.

### In situ hybridization

The samples were fixed overnight in DEPC-treated 4% paraformaldehyde at 4°C, cryoprotected in 30% sucrose overnight at 4°C, embedded in Tissue-Tek OCT 4583 compound and snap frozen. 10 µm sections were cut on a cryostat and transferred to Superfrost microscope slides (Fisher Scientific Co., Pittsburgh, Pennsylvania, USA). Digoxigenin-labeled probes were prepared according to the manufacturer's instruction (Roche Molecular Biochemicals, Mannheim, Germany). Probes used for in situ analysis were *Bmp4*, *Fgf8* (a kind gift from Dr. Janet Rossant, the Hospital for Sick Children, Toronto), *Wnt4* (a kind gift from Dr. Andy McMahon at Harvard University), and *Gli1* (a kind gift from Dr. Alexandra Joyner, Sloan Kettering Institute, New York). Details of the in situ analysis protocol may be obtained upon request.

## Results

### Generation of a *Tcf21-Cre* founder mouse line

**Figure 1:**
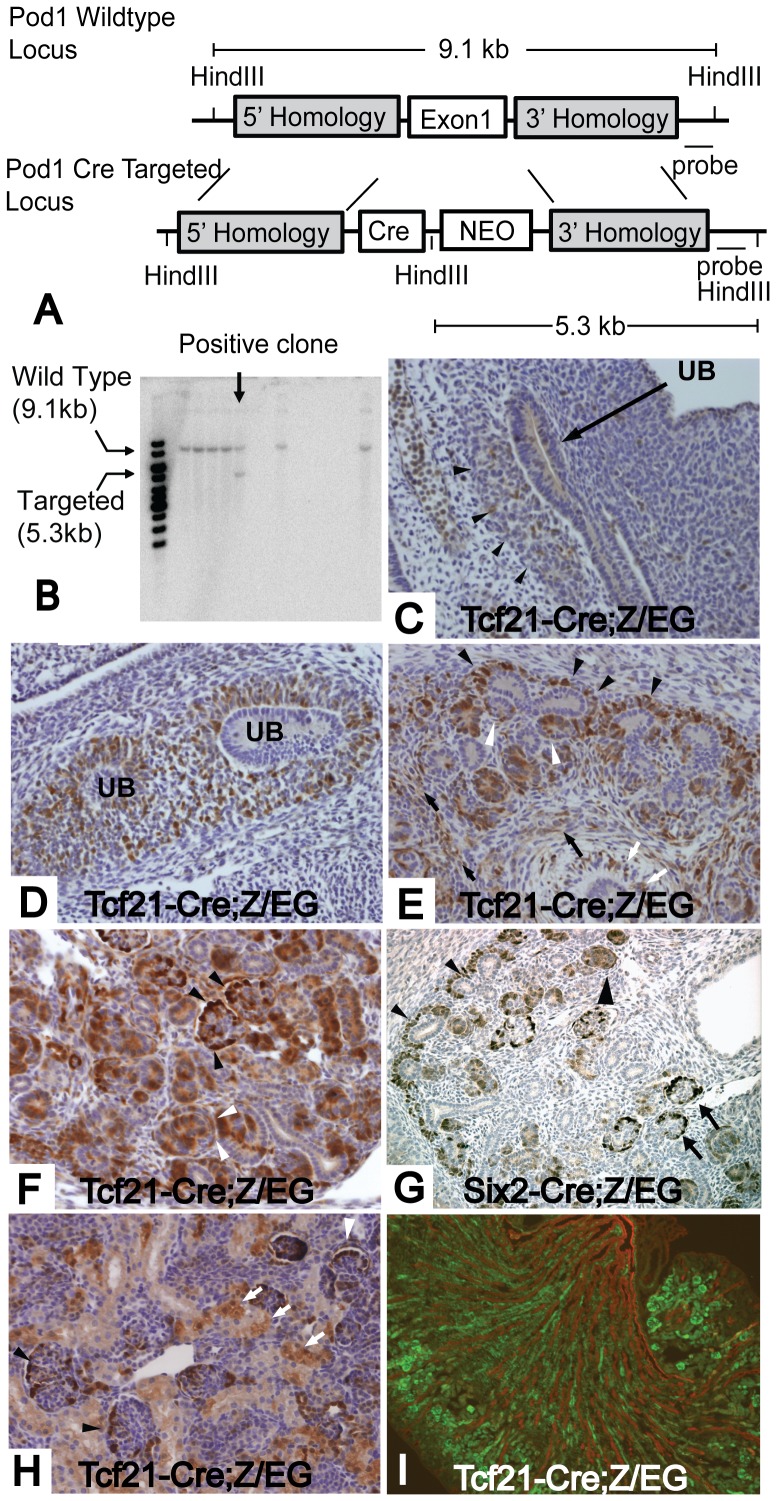
*Tcf21-Cre* mouse delete genes in metanephric mesenchyme and its derivatives. (A) Scheme of *Tcf21-Cre* targeted allele: Exon1 of the *Tcf21* gene was replaced by a Cre transgene and neomycin cassette. (B) Genomic DNA from ES cell clones was isolated and digested with HindIII. The 500 bps probe outside the region of 3′ homology arm recognized a 5.3 kb and a 9.1 kb fragment for the mutant and wild-type alleles, respectively. *Tcf21-Cre* (C–F and H, I) and *Six2-Cre* (G) mice were bred to the *Z/EG* reporter mousline and Cre dependent gene deletion was examined by immunostaining for GFP. (C) At E10.5, thin and mosaic expression of GFP is present in metanephric mesenchyme (black arrowhead). (D) E11.5 embryo shows distinct expression of GFP in metanephric mesenchyme. Ureteric bud is undergoing primary branching. (E) GFP expression is observed in condensing mesenchyme (black arrowhead), pretubular aggregates (white arrowhead), interstitial stromal cells (black arrow), mesenchyme surrounding the ureteric bud (white arrow) at E13.5 embryo. (F) At E16.5, GFP is expressed in developing nephrons and stromal cells. Staining in S shaped body (white arrowhead) and presumptive podocytes (black arrowhead) can be seen in this picture. (G) *Six2-Cre* gene deletion is restricted to condensing mesenchyme (black arrowhead), developing nephrons (large black arrowhead) and podocytes (black arrow) at E16.5. (H) At postnatal day 0, gene deletion in podocytes (black arrowhead) and Bowman's capsule (white arrowhead) is evident, but tubular staining was mosaic (white arrows). (I) P0 kidney was stained with GFP (green) and pancytokeratin (red). There was no overlap between the two stainings. Magnification: C–H 200×, I 40×.

The targeting construct for *Tcf21-Cre* was designed to replace the first exon of the *Tcf21* gene with Cre recombinase and a neomycin cassette (Fig. 1A). After electroporation, murine ES cell clones were screened by Southern blot analysis using a HindIII digest and 3′ probe outside the region of homology. One correctly targeted ES cell clone was used to generate chimeric mice that gave germline transmission (Fig. 1B).

### *Tcf21-Cre* driver mouse line excises genes in the metanephric mesenchyme and its derivatives

*Tcf21-Cre* mice were bred with *Z/EG* reporter mice. In embryonic day 10.5 metanephros, expression of the GFP reporter (denoting successful Cre-mediated excision) was observed in the condensing metanephric mesenchyme surrounding the invading ureteric bud (Fig. 1C). At this stage, GFP staining appeared to be thin and mosaic. At E11.5, GFP expression in metanephric mesenchyme was evident (Fig. 1D). By E13.5, gene deletion activity was shown in broad areas in the mesenchyme including condensing mesenchyme, developing nephrons such as pretubular aggregates and comma-shaped bodies, interstitial stromal cells, and the mesenchyme surrounding the stalk of ureteric bud (Fig. 1E). At E16.5, in addition to the GFP expression observed at E13.5, GFP expression was also appreciated in S-shaped bodies, immature podocytes in capillary loop glomeruli, and immature tubular cells (Fig. 1F). We compared the domain of *Tcf21*-driven Cre excision to that of the *Six2-Cre* mice. As seen in Fig. 1G, *Six2-Cre* activity is restricted to cap mesenchyme and its derivatives. At postnatal day 0, in *Tcf21-Cre;Z/EG* mice, GFP was observed in podocytes, tubules and cells of Bowman's capsule, though it appeared mosaic in the tubules (Fig. 1H). Double immunostaining for GFP and pancytokeratin showed no overlap, demonstrating that *Tcf21-Cre* is not active in collecting duct cells that are derived from the ureteric bud lineage (Fig. 1I). Taken together, *Tcf21-Cre* has the capacity for broad gene deletion in the mesenchyme and its derivatives, including both interstitial cells and epithelial components of the nephron. Thus, *Tcf21-Cre* is a valuable resource permitting the use of a single mouse Cre-driver strain to gain insight into multiple metanephric populations.

### *Tcf21-Cre* excises genes in mesenchymes in multiple organs

**Figure 2:**
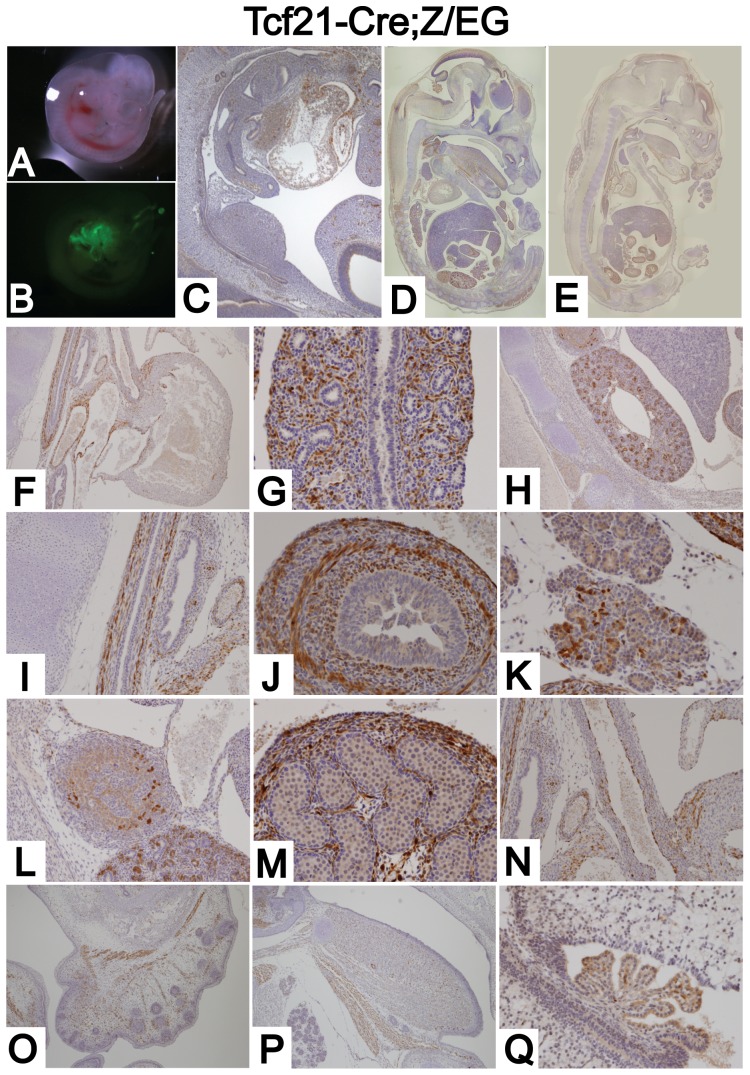
*Tcf21- Cre* mouse deletes genes in the mesenchyme of multiple organs. (A) Dissecting microscope photograph of E10.5 *Tcf21-Cre;Z/EG* embryo. (B) GFP expression of E10.5 *Tcf21-Cre;Z/EG* embryo was visualized by fluorescent microscopy. (C–Q) *Tcf21-Cre* mice were bred to a *Z/EG* reporter mouse line and Cre dependent gene deletion was examined by immunostaining for GFP. (C) E10.5 embryo shows GFP staining in heart and metanephric mesenchyme. (D) E13.5 and (E) E16.5 embryos show gene deletion in multiple organs. At E 16.5, GFP expression was observed in (F) some part of the epicardium and endocardium, (G) lung mesenchyme, (H) kidney mesenchyme, (I) proper muscle and lamina propria of esophagus, (J) proper muscle and lamina propria of intestine, (K) some part of pancreas, (L) adrenal gland, (M) stroma of gonad, (N) proximal part of aorta, (O) facial muscle, (P) lingual and sublingual muscle, (Q) choroid plexus of ventricles. Magnification: C 40×, F, H, O, P 100×, I, L, N 200×, G, J, K, M, Q 400×. Many 40× pictures were taken for D and E, and stitched together to construct the image of the whole embryo.

To explore the full potential of the *Tcf21-Cre* line, extrarenal Cre activity was assessed. At E10.5, GFP expression was observed in the 1^st^ to 3^rd^ branchial arches (Fig. 2 AB), the heart, and in metanephric mesenchyme surrounding the ureteric bud (Fig. 2C and 1B). E11.5 embryos showed a similar pattern (data not shown). At E13.5 and E16.5, GFP expression was prominent in epicardium, lung mesenchyme, kidney mesenchyme, lamina propria and smooth muscle layer of the gastrointestinal tract, pancreas, adrenal gland, gonads, proximal part of aorta, facial and sublingual muscles, and in the choroid plexus of the ventricles (Fig. 2D–Q).

### *Ctnnb1^fl/fl^*;*Tcf21-Cre* mice develop hydroureter and/or hypoplastic rudimentary kidneys

**Figure 3:**
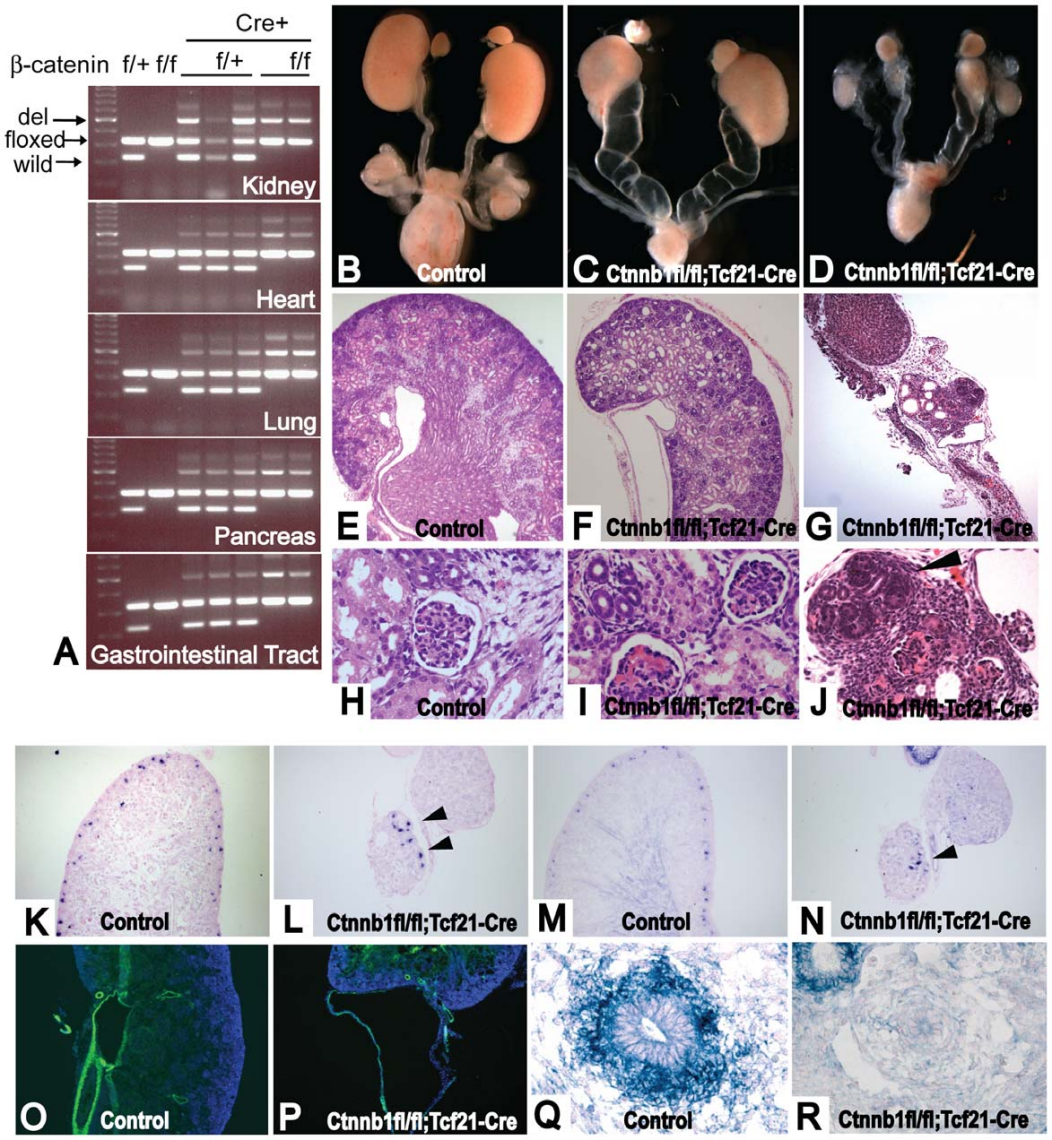
*Ctnnb1^fl/fl^;Tcf21-Cre* mice shows hydroureter or rudimentary kidneys. (A) DNA extracted from whole organs was subjected to PCR that detects wild type, floxed allele, and deleted allele of *β-catenin*. Deleted alleles were detected in kidney, heart, lung, pancreas and gastrointestinal tract. (B, E, H) Control kidney, (C, F, I) Hydroureter and relatively normal kidney of *Ctnnb1^fl/fl^;Tcf21-Cre* mice, (D, G, J) Rudimentary kidney and hypoplastic kidney with hydroureter of *Ctnnb1^fl/fl^;Tcf21-Cre* mouse. (F) Hydroureter kidneys had relatively normal appearance, but show dilation of tubules, (I) dilation of glomerular capillaries. Some of the glomeruli appear to be normal. (G) Rudimentary kidney was cystic, (J) had random nephrogenic area (black arrowhead) with a few disorganized glomeruli. (K) *Fgf8* expression in control kidney. (L) *Fgf8* is observed only in the disorganized nephrogenesis area (black arrowhead). (M) *Wnt4* expression in control kidney. (N) *Wnt4* is observed only in the disorganized nephrogenesis area (Black arrowhead). (O) Expression of *α*-*Sma* (green) in control mouse. Blue shows DAPI staining. (P) In hydroureter mutant, *α*-*Sma* (green) is present in the ureter wall, but its layer is extremely thin. (Q) *Bmp4* expression surrounding the ureteric bud in E13.5 control. (R) Expression of *Bmp4* surrounding the ureteric bud is lost in E13.5 *Ctnnb1^fl/fl^;Tcf21-Cre*. However, *Bmp4* expression is restored in developing nephrons. Experiments were performed on E18.5 embryos if not indicated. B–D are taken at the same magnification. Mice that lack at least one of the transgenes were used as controls. Magnification: E–G, K–P 100×, H–J 200×, Q, R 400×.

Wnt-β-catenin signaling plays a key role in the interaction between the metanephric mesenchyme and the ureteric bud during renal development [19], [20]. To validate the *Tcf21-Cre* line, we bred *Tcf21-Cre* mice with a β-catenin conditional loss of function (LOF) mouse (*Ctnnb1^fl/fl^*;*Tcf21-Cre*, referred to as β-catenin LOF mutant, hereafter). The β-catenin LOF mutants were born with the expected Mendelian ratio, but died within a few hours after birth. The deletion of *β-catenin* gene was confirmed by PCR, and was observed in the kidney and extrarenal tissues where Tcf21 is expressed including heart, lung, pancreas and gastrointestinal tract (Fig. 3A). Examination at E18.5 and in P0 pups demonstrated hydroureter with hypoplasia in 73.5% of the mutants, whereas 20.5% of mutants showed hypoplastic rudimental kidneys (Fig. 3B–D). Most of the mutant kidneys showed various degrees of hypoplasia. Kidneys with hydroureter demonstrated dilated tubules (Fig. 3F) and expansion of capillary loops, but some glomeruli appeared to be intact (Fig. 3I). The rudimentary kidneys were cystic, had only a few immature glomeruli, and showed random, disorganized nephrogenic aggregates at the periphery (Fig. 3GJ). As expected from this phenotype, expression of nephrogenic induction markers, *Wnt4* and *Fgf8*, were markedly reduced and only detectable in the disorganized nephrogenic area (Fig. 3K–N). Neither tubular structures nor interstitial space were present.

In order to highlight the unique characteristics of *Tcf21-Cre*, we compared the phenotype to that of previously published mice with *β-catenin* deletion in the cap mesenchyme (*Six2-Cre*) or stromal cell (*FoxD1-Cre*) populations. *Six2-Cre*;*Ctnnb1^fl/fl^* mice were deficient in nephrogenesis in the nephrogenic zone, whereas *FoxD1-Cre*;*Ctnnb1^fl/fl^* mice failed to develop the interstitial space [20], [21]. Compared to these compartmentalized phenotypes, the *Tcf21-Cre* phenotype extends from the nephrogenic zone, the interstitial space to the ureter, and also provides a range of severity of phenotypes among littermates due to a variable degree of mosaicism (Table 1). Together, these results demonstrate that the use of *Tcf21-Cre* allows the assessment of gene function in both the stromal and cap mesenchymes.

**Table 1:** Comparison of beta-catenin loss of function mutants by various renal Cre lines.

	*Ctnnb1^fl/fl^*;*Six2-Cre*	*Ctnnb1^fl/fl^*;*FoxD1-Cre*	*Ctnnb1^fl/fl^*;*Tcf21-Cre*
Nephrogenic Zone	Hypoplastic kidney and reduced nephron number. Lack of nephrogenic zone. No expression of inductive markers, such as Wnt4, FGF8 [20].	Relatively normal nephron induction [21].	Wide spectrum of hypoplastic kidneys, from almost normal to rudimentary kidneys. Reduced expression of inductive markers.
Interstitial space	None	Failure of medulla development	Failure of medulla development (Various)
Ureter	None	None	Hydroureter

In addition to the previously reported phenotypes, we observed a high incidence of hydroureter in β-catenin LOF mutants, likely a result of deletion in the periureteric bud mesenchyme that gives rise to smooth muscle cells. In keeping with this possibility, we examined the expression of α-Sma and *Bmp4* , which is known to play a key role in the development of the organized smooth muscle layer in the ureter [22], [23]. Although α-Sma positive cells were present in the dilated ureter wall, the smooth muscle layer was extremely thin (Fig. 3OP). Notably, *Bmp4* could not be detected in the mesenchyme surrounding the ureteric bud at E13.5 (Fig. 3QR), or in the dilated ureter at E18.5 (data not shown).

### *Ctnnb1^ex3/+^*;*Tcf21-Cre* mice develop fusion kidney or hypoplastic rudimentary kidney

Several recent papers have demonstrated that tight regulation of β-catenin expression is required for tissue homeostasis [24], [25]. Thus, as a second test to validate the *Tcf21-*Cre driver strain, we bred it with the conditional gain of function β-catenin mice (*Ctnnb1^ex3/+^*;*Tcf21-Cre*, referred to as β-catenin GOF mutant). Exon 3 of the mutant allele is flanked by loxP sequences. Upon Cre-mediated excision, the serine and threonine residues in exon 3 are removed, preventing phosphorylation and resulting in a stabilized β-catenin molecule that translocates and accumulates in the nucleus escaping ubiquitination and degradation [17].

**Figure 4:**
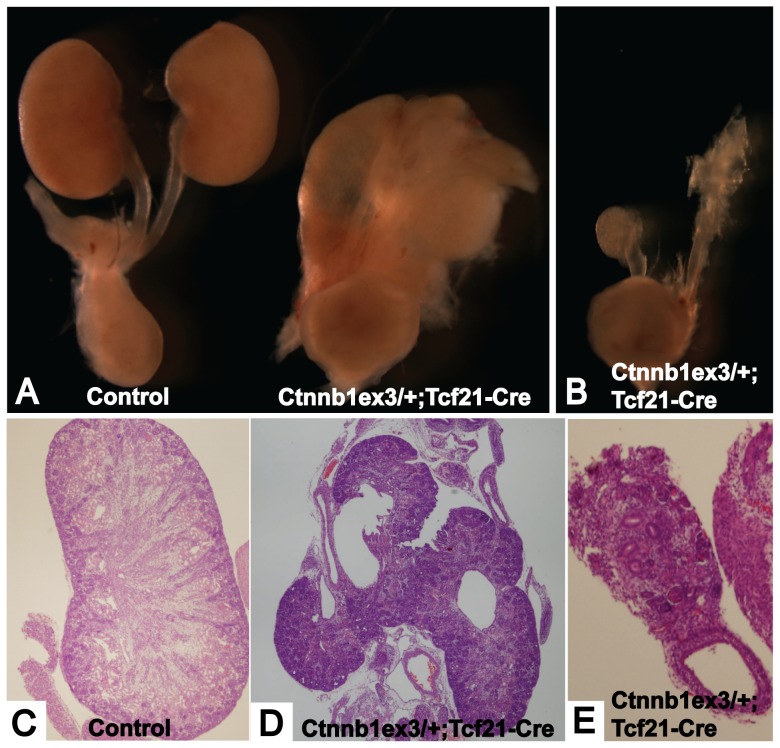
*Ctnnb1^ex3/+^; Tcf21-Cre* mice show fusion kidney and rudimentary kidney. (A) Control kidney (left) and fusion kidney of *Ctnnb1^ex3/+^;Tcf21-Cre* mouse (right). (B) Severely hypoplastic/dysplastic rudimentary kidney of *Ctnnb1^ex3/+^;Tcf21-Cre* mouse. (C) Normal histology of control kidney. (D) Histology of the fusion kidney. (E) Histology of the rudimentary kidney. Magnification: C 40×, E 100×. Many 40× pictures were taken for D, and stitched together to construct the image of the whole fusion kidney.

β-catenin GOF mutants die during embryonic life between E12.5 and P0 (data not shown). At E18.5, 66% of mutants demonstrated fusion kidneys at midline and 33% had severely hypoplastic/dysplastic kidneys with a few disorganized glomeruli (Fig. 4A–E). These phenotypes were striking and differed from the previously reported *Six2-Cre*;*β-catenin* gain of function phenotype, which is renal agenesis at birth [20]. Rudimentary kidneys of *Tcf21-Cre* mutants and agenesis observed in *Six2-Cre* mutants may both be due to failure of nephrogenesis in the cap mesenchyme and its derivatives, but midline fusion appears to arise as a result of the broader gene deletion of *Tcf21-Cre*.

Strikingly, 100% of the β-catenin GOF mutants also exhibited embryonic tumor that engulfed the kidney, gut, heart, and lungs, and appeared to arise from multiple mesenchymal tissues (Fig. S1). Importantly, this phenotype did not obscure the renal phenotype but as discussed below, provided new insight into possible genetic interactions between the β-catenin and Shh pathways.

### *Ptch1 ^fl/fl^*;*Tcf21-Cre* kidneys develop multiple renal cysts

As a final test of *Tcf21-Cre* line, we deleted *Ptch1*, the receptor for Shh. The Shh signaling pathway is crucial for the development of virtually all organs, and regulates cell fate determination, proliferation and tissue patterning [26]. After birth, Shh signaling is associated with certain types of malignancy [27], [28], [29]. *Shh* null mice show renal aplasia [30]. *Shh* and *Ptch1* deletion from ureteric bud result in hydroureter and hypoplastic kidney, respectively [31], [32]. However, the deletion of *Ptch1* in the metanephric mesenchyme has not been previously reported.

**Figure 5:**
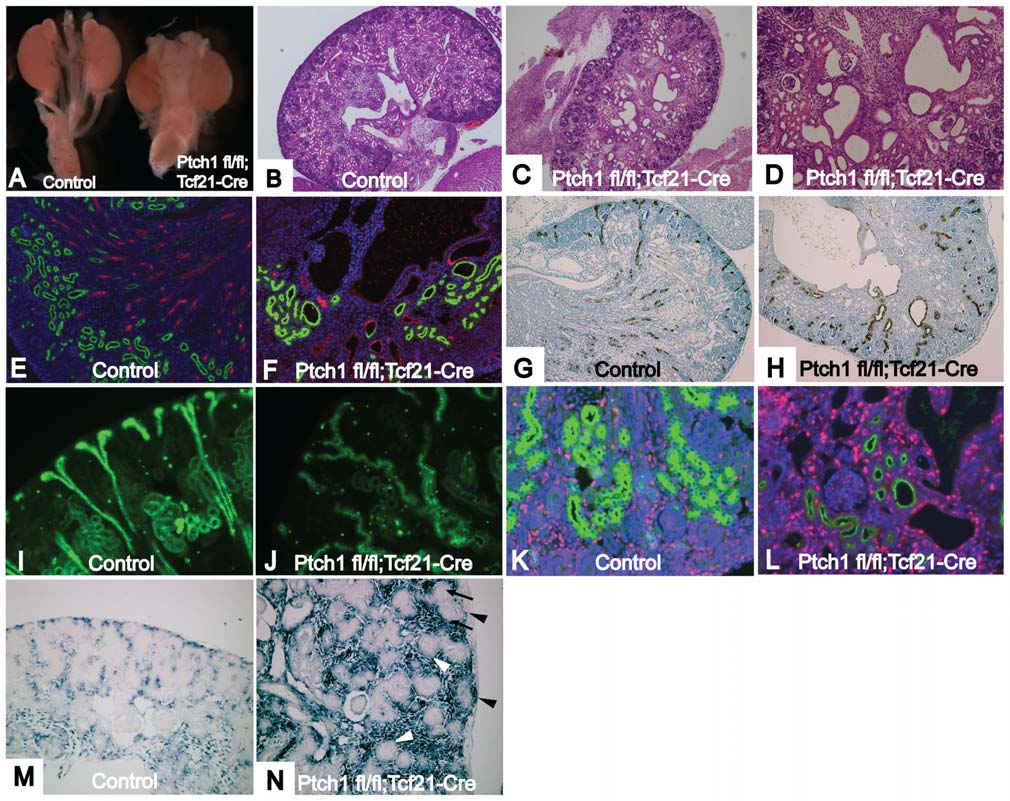
*Ptch1 ^fl/fl^;Tcf21-Cre* kidneys develop multiple renal cysts. (A) Control kidney (left) and *Ptch1 ^fl/fl^;Tcf21-Cre* kidney (right). (B) Histology of control kidney. (C) Multiple cysts of *Ptch1 ^fl/fl^;Tcf21-Cre* kidney. (D) Glomerular cysts as well as tubular cysts were observed in the mutant kidney. (E, F) Kidneys were stained with LTL-FITC (green) and THP (red) that mark proximal tubules or loop of Henle, respectively. (E) Control kidney shows beautiful organized structure of proximal tubules and loops of Henle. (F) Disorganized structure of proximal tubule and loop of Henle in*Ptch1 ^fl/fl^;Tcf21-Cre* kidney. Loops of Henle are very short. Some cysts were stained with either LTL or THP. (G–J) Sections were stained with DBA, which marks collecting ducts. (G, I) Control kidney show organized structure of collecting duct. Note the straight configuration of cortical collecting duct. (H, J) Structure of collecting duct system is severely disorganized in *Ptch1 ^fl/fl^;Tcf21-Cre* kidney. The path of collecting duct is random and winding. (K, L) Sections were stained with LTL (green) and Ki67 for proliferation. (K) normal kidney (L) *Ptch1 ^fl/fl^;Tcf21-Cre* kidney. Note that Ki67 is frequently positive in the cystic wall. (M and N) In Situ hybridization for *Gli1*, an established downstream target of *Shh* pathway. (M) Control kidney shows activation of *Shh* pathway in the interstitial area (N) Mutant kidney shows increased *Gli1* expression in condensing mesenchyme (black arrowhead) and stroma, but is negative in pretubular aggregates (black arrow) and nephrogenic vesicles (white arrowhead). Magnification: B, C 40×, D, G, H 100×, E, F, I–N 200×.

*Ptch1^fl/fl^*;*Tcf21-Cre* mice (referred to as *Ptch1* mutants) die at varying time points between E12.5 to E18.5 due to an aggressive embryonic tumor with striking similarities to the tumors observed in the β-catenin GOF mutants described above (Fig. S1). *Ptch1* mutant kidneys were of a similar size to those of *Ptch1^fl/fl^* littermates (Fig. 5A). Renal invasion of the tumor appeared to be minimal (Fig. 5C). Histological examination of E18.5 mutant kidneys demonstrated dilation of the pelvic area and multiple cysts (Fig. 5CD). In some cysts, glomeruli were observed, whereas other cysts arose in the renal tubules (Fig. 5D).

Stainings with Lotus tetragolonobus lectin (LTL), Tamm Horsfall protein (Thp), and Dolichos biflorus agglutinin (DBA) which marks the proximal tubules, loop of Henle, and collecting ducts respectively, showed that the cysts arose along the entire nephron (Fig. 5E–J). Mutant collecting ducts were dilated, distorted and winding (Fig. 5J). Additionally, immunostaining for Ki67 showed increased cell proliferation in the cystic wall, suggesting a possible mechanism for cyst formation (Fig. 5L).

We examined the expression of *Gli1*, a well-established marker of Shh activation. As *Ptch1* suppresses the Shh pathway, genetic deletion of the *Ptch1* gene should result in upregulation of *Gli1* expression. In keeping with this, in situ hybridization showed increased expression of *Gli1* in the condensing mesenchyme and interstitial cells at E16.5 (Fig. 5N). However, surprisingly, *Gli1* expression was not seen in developing nephrons, such as pretubular aggregates and S-shaped bodies.

## Discussion

Here we report the generation of a novel Cre transgenic mouse line, *Tcf21-Cre*, that allows gene excision in the metanephric mesenchyme and its derivatives, including interstitial cells and all epithelial components of the nephron from podocytes to distal tubules. It also results in gene deletion throughout the mesenchyme of other organs including lung, heart, gastrointestinal tract, pancreas, gonad and adrenal gland. Gene deletion begins at E10.5 in the condensing metanephric mesenchyme, and is consistently observed in its derivatives throughout renal development.

Currently, *Six2-Cre* and *Pax3-Cre* are most widely used to investigate the role of genes in the developing kidney and metanephric mesenchyme [4], [6], [7]. Is there any advantage or need for *Tcf21-Cre*? In contrast to *Tcf21*, *Six2-Cre* expression is restricted to the cap mesenchyme. While this is useful to determine gene function in this specific compartment, *Tcf21-Cre* provides a broader mesenchymal excision from both stromal and cap compartments as well as mesenchymal cells that give rise to periureteric bud smooth muscle cells. Similar to *Six2-Cre*, *Tcf21-Cre* expression remains active in epithelial nephric derivatives but unlike *Six2*, *Tcf21* is also expressed in interstitial lineages. As a first step in validation, we compared and contrasted phenotypes in mice lacking β-catenin following *Six2* versus *Tcf21-Cre* deletion. As expected, we observed many similarities but also important differences. Given the broader expression domain of *Tcf21*, we observed hydroureters and a smooth muscle cell phenotype with down-regulation of *Bmp4*. Similarly, gain of function studies of *β-catenin* provided similarities but also some differences, with *Tcf21-Cre* causing midline fusion of the kidneys in β-catenin GOF mutants.

How does *Tcf21-Cre* compare to the *Pax3-Cre*? Similar to *Tcf21-Cre*, *Pax3-Cre* reportedly excises in both epithelial and interstitial lineages that derive from cap and stromal metanephric mesenchyme. However, the extrarenal sites of Pax3-Cre activation are largely different, and include derivatives of neural crest and somites, such as dorsal root ganglia, skeletal muscle, adrenal medulla, and some subsets of colon epithelium, which do not overlap with the mesenchymal distribution of *Tcf21-Cre* expression [6], [7].

An additional benefit of *Tcf21-Cre* includes the method of generation of the Cre line through homologous recombination in ES cells. This allows faithful recapitulation of expression pattern ensuring stable expression over subsequent breedings. Randomly inserted or BAC transgenic lines may or may not provide this asset. It is worthwhile to note that *Tcf21* is not expressed in intermediate mesoderm [33], [34], which suggests that the GFP expression of *Tcf21-Cre; Z/EG* mice doesn't include an excision prior to the development of metanephros. In addition, we noted mosaicism of the *Tcf21-Cre* expression and excision. Although this might be viewed as a negative (incomplete excision), we found it to be an asset in our experiments. As demonstrated with our proof of principle studies in this report, a wide spectrum of phenotypes can be observed in each litter ranging from severe to mild, allowing a more complete picture of gene function– i.e. permitting a dose response experiment in a single litter. Finally, the extrarenal expression of *Tcf21* in other mesenchymal tissues allows insight from other organs. Although extrarenal expression of a Cre driver strain is often viewed as a weakness, it may provide additional information for the investigator. In this study, although we observed robust extrarenal phenotypes in all of the mutants, they did not preclude analysis of phenotype in kidney. One of the reasons for this is the relatively late onset of Tcf21 expression at mid-gestation. In turn, the development of aggressive embryonic sarcomas in both β-catenin GOF and Ptch1 LOF mutants points to a common genetic pathway – providing direction for future studies.

Utility of *Tcf21-Cre* strain is further underscored by the phenotypes observed in the *Ptch1* mutants. Although a role for *Shh* pathway in renal cystic diseases is supported by dysregulation of *Gli1* in nephronophthisis , there has not been any direct genetic evidence [35], [36]. Here, we show the development of a dramatic cystic renal phenotype in mice lacking the *Ptch1* receptor with data to support a role for proliferation. Interestingly, we did not detect Shh pathway activation in the developing nephrons of *Ptch1^fl/fl^*;*Tcf21-Cre* mice. It remains to be determined whether the nephrons found in the mutant kidney are derived from wild type cells that escape Cre excision due to the inability of *Ptch1* null cells to form nephrons.

In summary, we report a new Cre driver strain that provides robust excision in the metanephric mesenchyme. It provides researchers with a useful tool to delete genes broadly from multiple metanephric mesenchyme subpopulations, resulting in phenotypes in the stromal cells, the cap mesenchyme and the periureteric bud cells.

## Supporting Information

Figure S1 ***Ptch1^fl/fl^;Tcf21-Cre*** **and** ***Ctnnb1^ex3/+^;Tcf21-Cre*** **mice show strikingly similar sarcomas.** Hematoxylin and eosin staining of (A, D, G, J) Control, (B, E, H, K) *Ptch1^fl/fl^;Tcf21-Cre*, (C, F, I, L) *Ctnnb1^ex3/+^;Tcf21-Cre*. (A–C) lungs, (D–F) liver, (G–I) gastrointestinal tract, (J–L) pancreas. The sections were examined by two experienced pathologists and diagnosed as sarcomas, which invade multiple organs. Magnification: all 40×.
(TIF)
