# Supplementary figures and images for: A New Cre Driver Mouse Line, Tcf21/Pod1-Cre, Targets Metanephric Mesenchyme
Source: PLoS One. 2012 Jul 6;7(7):e40547. doi: 10.1371/journal.pone.0040547 (PMC3391250; doi:10.1371/journal.pone.0040547)

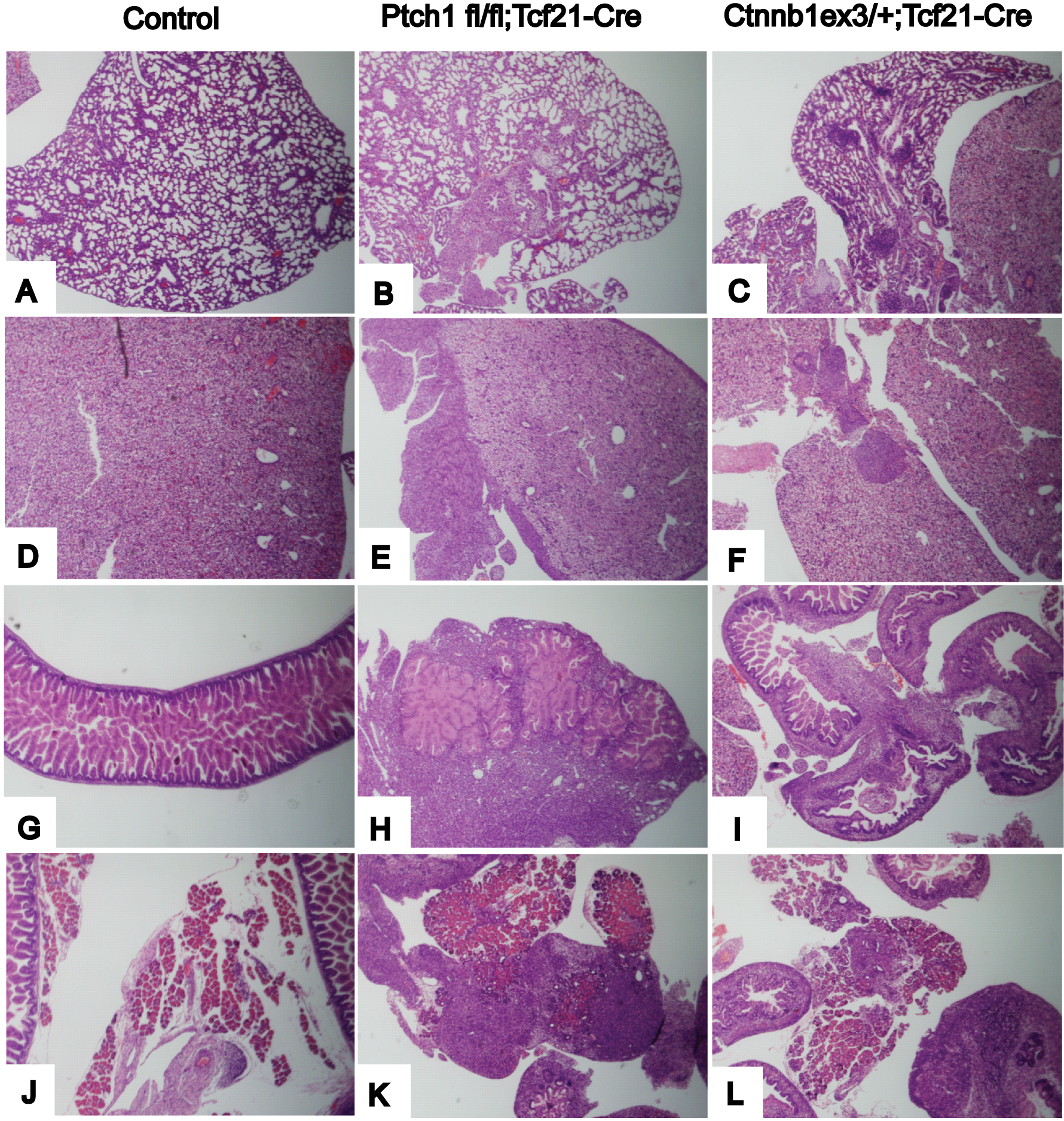

Supplement: Figure S1 — Ptch1fl/fl;Tcf21-Cre and Ctnnb1ex3/+;Tcf21-Cre mice show strikingly similar sarcomas. Hematoxylin and eosin staining of (A, D, G, J) Control, (B, E, H, K) Ptch1fl/fl;Tcf21-Cre, (C, F, I, L) Ctnnb1ex3/+;Tcf21-Cre. (A–C) lungs, (D–F) liver, (G–I) gastrointestinal tract, (J–L) pancreas. The sections were examined by two experienced pathologists and diagnosed as sarcomas, which invade multiple organs. Magnification: all 40×. (TIF) [file pone.0040547.s001.tif]
